# Islet Cell Tumour of the Pancreas

**Published:** 1956-10

**Authors:** T. F. Hewer


### ISLET CELL TUMOUR OF THE PANCREAS

(*A Clinical Pathological Conference of the University of Bristol Medical School.*)

CHAIRMAN: PROFESSOR T. F. HEWER

*Dr. A. D. McInnes:* This is the case of a young housewife aged 25, admitted under the care of Dr. Birrell on the 19th August, 1955, in an unconscious state. The history obtained from the husband was that of a 2½-year history of attacks of unconsciousness. At the beginning of her illness, in February 1953, she did not actually lose consciousness but behaved in a peculiar manner for up to two hours, for which period she had complete amnesia. After a few weeks she began to lose consciousness in the attacks and they lasted up to five or six hours. They occurred at no particular time of the day and no conducive circumstances were mentioned by the husband.

She was seen at an institute in April 1953 and a diagnosis of idiopathic epilepsy was made. An abnormal E.E.G. was noted. Anti-convulsive treatment was begun. The attacks continued despite treatment with epanutin, and luminal was added without much effect.

She was admitted to the Bristol Royal Infirmary on 10th February, 1954, at 11 p.m., having been in coma since 4 p.m. that day. She slowly regained consciousness spontaneously, was discharged the following day with a diagnosis of post-epileptic stupor and was referred to the Institute where she was receiving treatment.

These attacks increased in frequency to such an extent that termination of a three months' pregnancy was advised by the Institute and hysterotomy was accordingly carried out in March 1954. Thereafter her attacks occurred only once every second month until August 1954 when the frequency increased and epanutin was changed to hydantil.

She was finally admitted to the Bristol Royal Infirmary at 9 p.m. on 19th April, 1955, having been unconscious for 24 hours. On admission her temperature was 103° F., all limbs were flaccid but moved in response to painful stimuli, the plantars were both flexor and fundi normal. No diagnosis was made at that stage and the patient was started on penicillin. The following morning tachycardia and pyrexia persisted. Respirations slightly increased and her skin was hot and dry. She no longer responded to painful stimuli; the left plantar response was extensor and the right equivocal; the right pupil was slightly larger than the left, but pupil reactions were normal; the fundi were normal. Although there was no clinical evidence of increased intracranial pressure, in view of the bizarre story of epilepsy it was considered she might have psychomotor epilepsy as the result of a space-occupying lesion in the right temporal lobe. A neurosurgical opinion was therefore sought and she was seen by Mr. Alexander that afternoon. He described the patient as barely rousable but able to move her limbs in response to painful stimuli, the left more than the right. There was a left plantar extensor, the right probably so. The fundi were normal. 40 c.c. of 50 per cent. dextrose were administered by Mr. Alexander intravenously "with only a fleeting lightening of stupor". It was concluded that acute hydrocephalus and hypoglycaemia had been excluded. Lumbar puncture was recommended and performed with normal results of pressure, protein and cells.

She was seen later that evening by Dr. Crow who considered that spontaneous hypoglycaemia was still a possibility but did not press for an immediate blood-sugar. When this was done the following morning it was found to be 10 mg. per cent. Hence the cause of her coma was revealed and with it the probability that her whole illness was due to a pancreatic islet cell tumour. She was given 150 c.c. of 50 per cent. dextrose intravenously and then 700 c.c. of 10 per cent. dextrose drip in 2½ hours. After this the blood-sugar did reach 210 mg. per cent. Despite this there was no change in the patient's condition. Respirations became irregular; the blood-pressure fell; she became pulseless. She was put in a respirator but she did not recover. A diagnosis of pancreatic islet cell tumour was made before she died.

*Mr. G. L. Alexander:* I was called in because of the unconsciousness and something of a neurological history. As far as I could work out from the glucose given (40 c.c. of 50 per cent). This would raise the blood-sugar 17 mg. per cent. and this would leave her with only 27 mg. per cent. which would still be low. The lesson that I have learnt is that a large dose of glucose must be given in these cases. I hesitated to call out the lab. on Saturday afternoon. There is some doubt whether even if this treatment had been given on admission she would have recovered. The brain sometimes appears to be intolerant of low blood-sugar leading to—no, not coma, but—heavy stupor. In other cases unconsciousness can last 48 hours or more and spontaneous recovery still occur. Some cerebra can tolerate low blood-sugar for a considerable time.

*Student:* Had the attacks any relationship to food, from the clinical history?

*Dr. A. D. McInnes:* The husband was a very accurate observer, making a careful note of the times and length of attacks. They were very rarely in the morning, only one had occurred before breakfast. They were mostly between 3 and 5 o'clock in the afternoon.

*Professor T. F. Hewer:* At what age did the first attack occur?

*Dr. A. D. McInnes:* The first attack was in February 1953.

*Professor T. F. Hewer:* She was then 22 or 23, and never before classed as epileptic, which is very atypical of idiopathic epilepsy. Let us have the post-mortem before we go any further.

*Dr. R. L. Bishton (pathologist):* Yes this was a case of an islet cell tumour. A young woman externally apparently healthy apart from a hysterotomy scar and a purpuric rash in the axilla. There was collapse of the lungs. I had fortunately heard the history of low blood-sugar so I went straight to the pancreas. I sliced through the pancreas. There was a hard nodule there, not difficult to find, 6 inches from the duodenum, whitish pink in colour. Histologically it had a rather acinar appearance but was certainly islet cell tissue.

Several conditions can give a picture of hyperinsulinism. It is all right to diagnose it and then ask the surgeon to look. Sometimes there are multiple adenomata; sometimes the tumour is very easy to feel because of a lot of fibrous tissue; sometimes there is just a hyperplasia of the islets and sometimes the tumour can be of the same consistency as the rest of the pancreas. Sometimes the pathologist is at a loss, as in the case of a young boy who was a patient of Professor Milnes Walker. I could find nothing but the boy recovered after operation so it must have been there. Here the surgeons would have been lucky had she been seen much earlier. It is possible to have adenomata of the pancreas with or without hypoglycaemia. The islet cell tissue is derived by a budding-out process from the rest of the pancreas, and finally produces insulin. I read a recent article in the *Journal of Pathology and Bacteriology* on adenoma of the pancreas (Spencer H. (1955) *J. Path. and Bact.*, **69**, 250). Some adenomata secrete insulin. Some differentiate and others do not. Some are palpable. This one was easily palpable. The brain showed a little oedema.

*Professor T. F. Hewer:* Perhaps Mr. Alexander would like to say a word?

*Mr. G. L. Alexander:* I do not know what to say about this. This is the second case of islet cell adenoma that has come our way previously diagnosed as epilepsy, both on the grounds of electroendephalography. There are lots of nice machines nowadays which do not give a straight answer. Some epileptics have epilepsy and give no abnormal pattern while others give an abnormal pattern and do not exhibit epilepsy. I am a bit of an iconoclast about these instruments. They are an extremely useful guide to the presence of an abscess or tumour of the frontal lobe, and a guide to treatment for combination of symptoms. I always judge by the fit. The E.E.G. is just confirmatory evidence; the fit is the much more telling bit of evidence.

*Dr. R. L. Bishton:* There was an interesting case of a dancing instructor frequently in trouble with the police for supposed drunkenness. Fortunately he was spotted, and his illness was found to be due to hypoglycaemia. He is now all right.

*Dr. E. J. Field:* Was there any evidence of pituitary change?

*Dr. R. L. Bishton:* I did not note this microscopically.

*Professor T. F. Hewer:* Would Dr. Norman describe the changes in the brain for us?

*Dr. R. M. Norman:* The distribution of the cerebral changes in hypoglycaemia are much the same as in anoxic conditions. In severe cases there may be widespread necrosis of nerve cells in the cerebral cortex, often in a roughly laminar pattern. The cornu Ammonis, especially its Sommer sector, is particularly vulnerable and the corpus striatum and cerebellar cortex are also favoured sites for severe destruction. In some cases a selective degeneration of the granular layer of the cerebellum has been reported but usually it is the Purkinje cells which suffer most. In one case of islet cell hyperplasia described a few years ago there was a marked reduction in the number of anterior horn cells in the spinal cord in addition to the more usual findings. There had been conspicuous muscle wasting in the hands and feet. Although these cerebral changes may be aggravated by the effects of the convulsions or of circulatory failure it is generally agreed that they are essentially due to deprivation of glucose. It is therefore not surprising that there is a close resemblance to what is found in anoxia. It is quite possible for acute nerve cell changes to occur in a few hours. The cells lose their Nissl bodies, the cytoplasm stains pink with eosin and the nucleus becomes triangular and pyknotic. This co-called "ischaemic nerve cell change" is common in anoxic conditions and similar changes occur after hypoglycaemia, though the nucleus may be more obviously damaged. This ischaemic cell degeneration has been known to appear within half an hour in a man who committed suicide by hanging.

*Question:* Do these changes occur in about 48 hours with hypoglycaemia?

*Dr. R. M. Norman:* Yes, but I do not know whether they can come on so quickly as in anoxia.

*Dr. B. E. McConnell:* The interpretation of these E. E. G. changes seems to have had far-reaching implications. I have several epileptics in my own practice, and two at least have had children. There is one who is pregnant. She has been seen by every psychiatrist in Bristol. She is, I think, the only person to be banned from Barrow Mental Hospital for bad behaviour! She is also an hysteric. She threw a fit one Sunday evening at a prayer meeting and I was called to take her away. This I did quite simply by telephoning the St. John Ambulance. The driver asked me the name of the patient and when I told him he said, "Oh no, not her!"

With one year's history of epilepsy what were the indications for hysterotomy?

*Dr. A. D. McInnes:* It was on the neurologist's decision that it was terminated.

*Dr. J. A. Cosh:* Damage done to the brain by hypoglycaemia and by anoxia is sometimes reversible. I remember two examples: The first was a very carefree character, a diabetic, who took his insulin one morning without eating breakfast and then while he was driving in Somerset he felt dizzy and ill. He managed to stop the car, struggled out, gave himself more insulin and collapsed. He was picked up by an ambulance and sent to the B.R.I. We gave him intravenous glucose and a good meal in Casualty, and returned him home later in the day when he had recovered. Whereupon he was immediately returned to hospital with an irate note from his doctor asking was this how we treated hypogloycaemics in Bristol. He was in a dazed, stuporose condition, in spite of intravenous glucose and it took 4 days to get him back to normal although he had a raised blood-sugar and was passing sugar in his urine during this time.

*Professor T. F. Hewer:* He was quite all right afterwards?

*Dr. J. A. Cosh:* Yes. The other was an example of cerebral anoxia. She had mitral stenosis and auricular fibrillation and was to have her teeth out. She went to the theatre. As the endotracheal tube was being passed, before she was deeply under the anaesthetic, her heart stopped beating for 5–6 minutes. An abdominal incision was made for cardiac massage when her heart started beating again, now in a normal rhythm. When she came round from what anaesthesia she had had she was in a dazed, disorientated conditions for three days before she returned to normal and she appeared to have loss of memory for that period.

*Student:* Following this did her heart go on beating in a normal rhythm?

*Dr. J. A. Cosh:* No; she developed auricular fibrillation again.

